# Probing the structure and electronic properties of beryllium doped boron clusters: A planar BeB_16_^−^ cluster motif for metallo-borophene

**DOI:** 10.1038/s41598-019-50905-7

**Published:** 2019-10-07

**Authors:** Dongliang Kang, Weiguo Sun, Hongxiao Shi, Cheng Lu, Xiaoyu Kuang, Bole Chen, Xinxin Xia, George Maroulis

**Affiliations:** 1 Institute of Atomic and Molecular Physics, Sichuan University, Chengdu, 610065 China; 2 School of Mathematics and Physics, China University of Geosciences (Wuhan), Wuhan, 430074 China; 3 Department of Chemistry, University of Patras, GR-26500 Patras, Greece

**Keywords:** Structural properties, Electronic properties and materials

## Abstract

Beryllium-doped boron clusters display essential similarities to borophene (boron sheet) with a molecular structure characterized by remarkable properties, such as anisotropy, metallicity and high conductivity. Here we have determined low-energy structures of BeB_*n*_^0/−^ (*n* = 10–20) clusters by utilizing CALYPSO searching program and DFT optimization. The results indicated that most ground states of clusters prefer plane or quasi-plane structures by doped Be atom. A novel unexpected fascinating planar BeB_16_^−^ cluster with C_2*v*_ symmetry is uncovered which possesses robust relative stability. Furthermore, planar BeB_16_^−^ offers a possibility to construct metallo-borophene nano-materials. Molecular orbital and chemical bonding analysis reveal the peculiarities of BeB_16_^−^ cluster brings forth the aromaticity and the strong interaction of B-B σ-bonds in boron network.

## Introduction

Molecular geometric configuration and attributes of pure^1–3^ and doped boron clusters^4–9^ have drawn much attention in recent years. The use of boron clusters as subunits in novel bioactive architectures with potential use as drugs is of primary importance^10^. From a Materials Science perspective the emergence of graphene^11^ and synthetic two-dimensional structures as silicene^12,13^, germanene^14^, stanene^15^, antimonene^16^, bismuthene^17,18^ and tellurene^19^ have opened new pathways for modern research^20–22^. Relying on experimental and theoretical work, Hersam’s group^23^ have confirmed and established the synthesis of 2D boron polymorphs (borophene) characterized by anisotropy and metallicity, and paved the way to investigations leading to the discovery of novel materials. Recently, it was reported that magnesium diboride (MgB_2_), which consists of graphene-like honeycomb networks of sandwiched boron, shows superconductivity^24^. It should be noted that beryllium has the same valence electrons number with magnesium. Be-doped boron clusters appear to have significant potential candidate as layered 2D materials^25–28^. This certainly gives reason for more systematic investigations.

Boron is the lightest metalloid chemical element, the lowest-Z element^23^ with a trivalent outer shell^29,30^. Consequently, boron does not form closed-shell electronic structures via conventional covalent bonds^31–33^, but favors delocalized chemical bonds with electron pairs shared among three (or more) atoms instead. Recently, systematic investigations of pure boron clusters in term of the anisotropy and polymorphism have brought forth new significant findings leading to the design of new borides. A selection of characteristic architectures of pure boron clusters includes: tank tread^34^, wankel motor^35–39^, wheel-like^40^, boron nanotubes^41^, B_12_ icosahedra^42^, buckyballs^43^, fullerene^44^, B_36_ with hexagonal holes (HHs)^45^, naphthalene^46^, borospherene^47^ and more. Co and Rh doped B_12_^−^ clusters featured half-sandwich structure has been reported by Wang and co-workers^48^. There followed the Co-centered boron molecular drums structure for the CoB_16_^−^ cluster^49^. Additional work by the same group includes the Mn-centered tubular boron cluster for MnB_16_^−^, a drum and quasi-planar structure for RhB_18_^−^ and the planar CoB_18_^−^^50–52^. Very recently, Cui and co-workers reported tubular structures for LiB_20_ and LiB_20_^−^^53^. These impressive findings reveal that single metal atom doping leads to new opportunities for the use of boron clusters as geometrical ligands.

Several theoretical investigations of boron clusters with doping transition-element serve as the object of discovering new materials recently^54,55^. The alkaline-earth metal-doped boron clusters and Be-doped ones in particular have been systematically studied^56–58^. Nevertheless, more systematic work is needed to systematize and deepen our understanding of Be-doped boron clusters. To fill the existing lacunae and bring forth new insights on medium-sized Be-doped boron clusters, we have thoroughly investigated BeB_*n*_^0/−^ clusters.

## Results and Discussion

### Geometric configurations and photoelectron spectra

**Figure 1:**
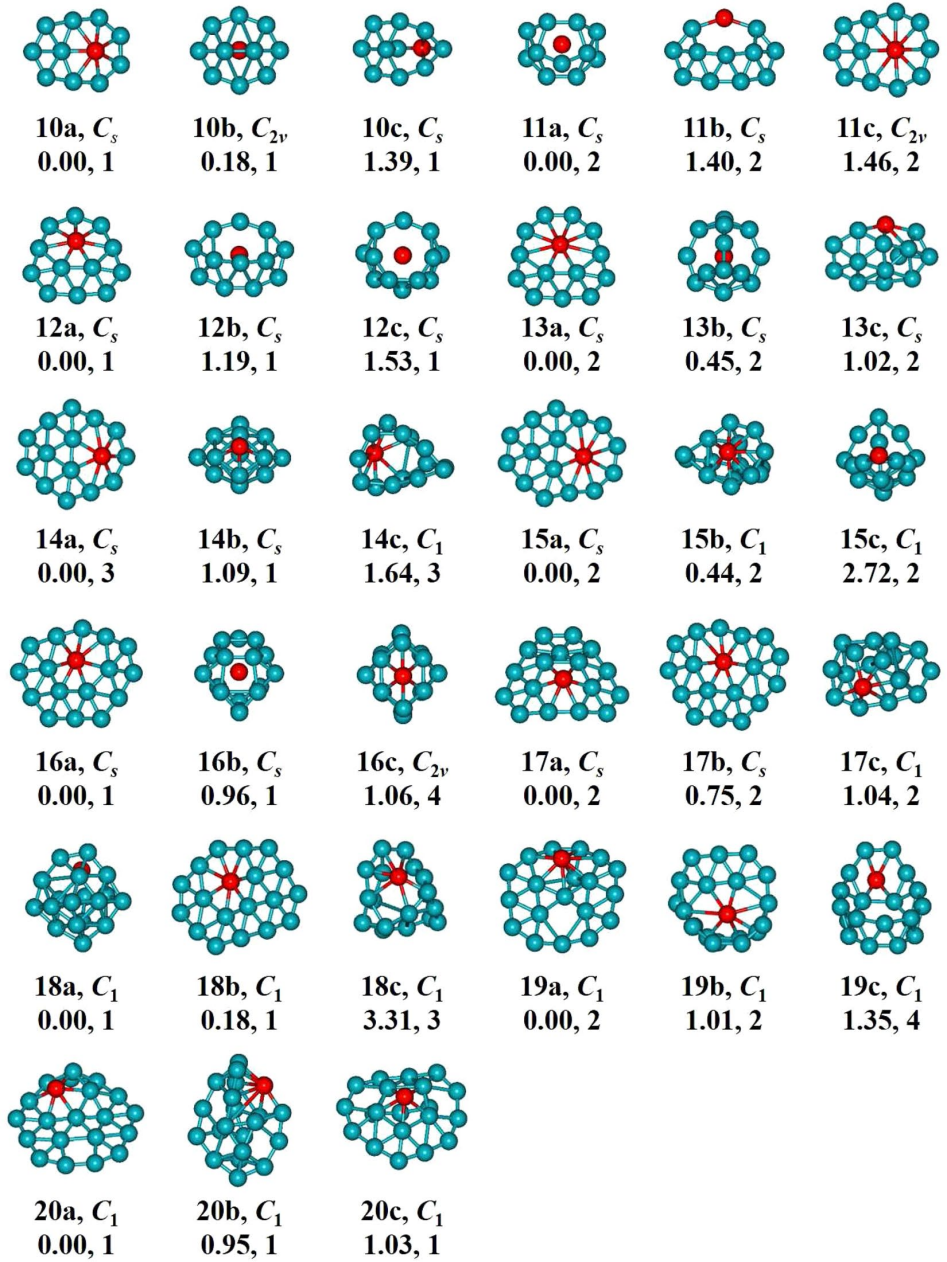
Low-lying geometrical structures of BeB_*n*_ (*n* = 10–20) clusters. “a” stands for the lowest-energy structures. “b” and “c” stand for the metastable state structures.

**Figure 2:**
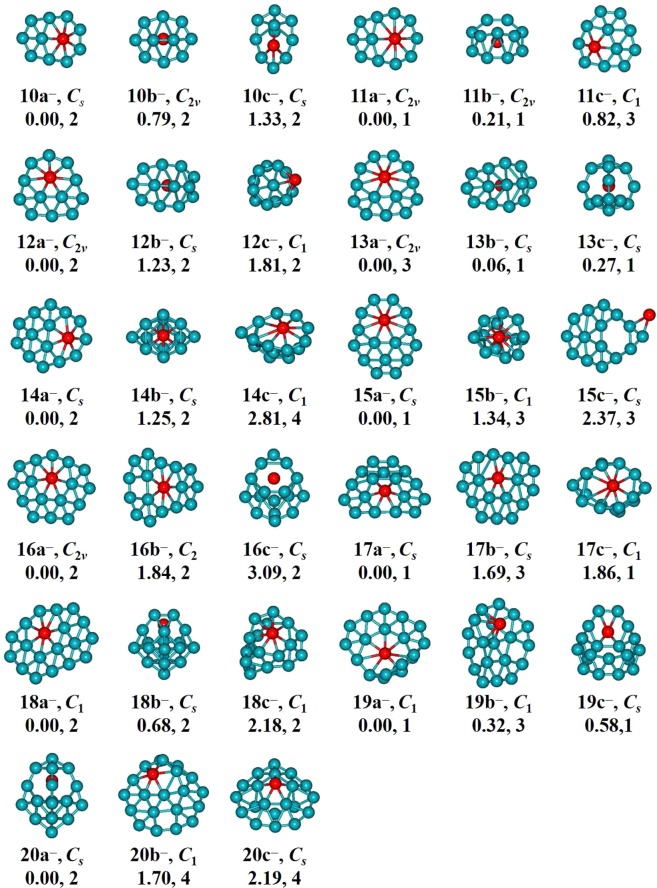
Low- lying geometrical structures of BeB_*n*_^−^ (*n* = 10–20) clusters. “a” stands for the lowest-energy structures. “b” and “c” stand for the metastable state structures.

The determined low-energy BeB_*n*_^0/−^ (*n* = 10–20) are showed in Figs 1 and 2. We labeled each isomer using *n*t/t^−^ (t = a, b, c), therein *n*t stands for the neutral clusters and *n*t^−^ stands for the anionic clusters. The lowest-energy structures BeB_*n*_^0/−^ (*n* = 10, 12, 13, 14, 15, 16) and BeB_11_^−^ are quasi-planar structures. The lowest-energy structure BeB_11_ shows a half-sandwich structure consisting of one half-sandwich structure composed by eleven boron atoms and one Be atom in the center. The lowest-energy structures BeB_17_^0/−^ like a trapezoid and its center portion appear on the convex. The lowest-energy structure of BeB_18_ and BeB_20_^−^ are 3D cage-like structure. The lowest-energy structure BeB_18_^−^ with a parallelogram located in the center displays a planar structure. The ground-state structures BeB_19_^0/−^ and BeB_20_ can be viewed as plate-like structures (in Figs S4 and S5 of Supplementary Information). The lowest-energy structures of BeB_*n*_^0/−^ clusters are generally evolutional from the quasi-planar to 3D cage-like or plate-like structures. For plane and quasi-planar structures, the coordinate number of Be atom is interesting. The BeB_*n*_^0/−^ (*n* = 10, 12, 14, 16) and BeB_18_^−^ feature heptacoordinate and the BeB_11_^−^ and BeB_*n*_^0/−^ (*n* = 13, 15) possess octacoordinate, while the BeB_17_^0/−^ are quasi-planar hexacoordinate structures due to the attribute of Be atom^59,60^. This evident structures evolution pattern contributes to form plane clusters of BeB_*n*_^0/−^, which are potential two-dimensional material. The metastable of *n*b/b^−^ (*n* = 10–13) clusters display half-sandwich architectural feature, while when the cluster sizes increase *n* ≥ 14, the clusters are varies cage-like, quasi-planar and plate-like structures. The *n*c/c^−^ (*n* = 10–18) clusters display half-sandwich, plane, cage-like structures, different from the larger size isomers (*n* ≥ 19) are double-ring and plate-like structures.

To get a deep understand to differences between different metal-doped clusters, we provide a comparison for doped boron clusters. The transition-metal doped boron clusters, NbB_10_^−^ and TaB_10_^−^, are wheels structures with high coordination number^4^, while BeB_10_^0/−^ clusters are quasi-planar structures with one B-Be unit inside. For doped B_12_ clusters, the prior works report that half-sandwich structures VB_10_^−^, CoB_12_^−^ and RhB_12_^−^ clusters^4,48^ are different with BeB_12_^0/−^ clusters, which are standard quasi-planar structures featuring a triangle in the center. Compare with drum-like CoB_16_^−^ cluster^49^ and tubular-like MnB_16_^−^ cluster^50^, the ground state BeB_16_^0/−^ display quasi-plane structures. It is worth noting that adjacent alkali element Lithium doped into B_20_ display highly symmetrical tubular LiB_20_^0/−^ clusters^53^. We report BeB_20_ and BeB_20_^−^ are plate-like and 3D cage-like structures, respectively. The reason for the structural differences of same-sized clusters may be doped-metals have different valence electron and atomic radius^61^.

**Figure 3:**
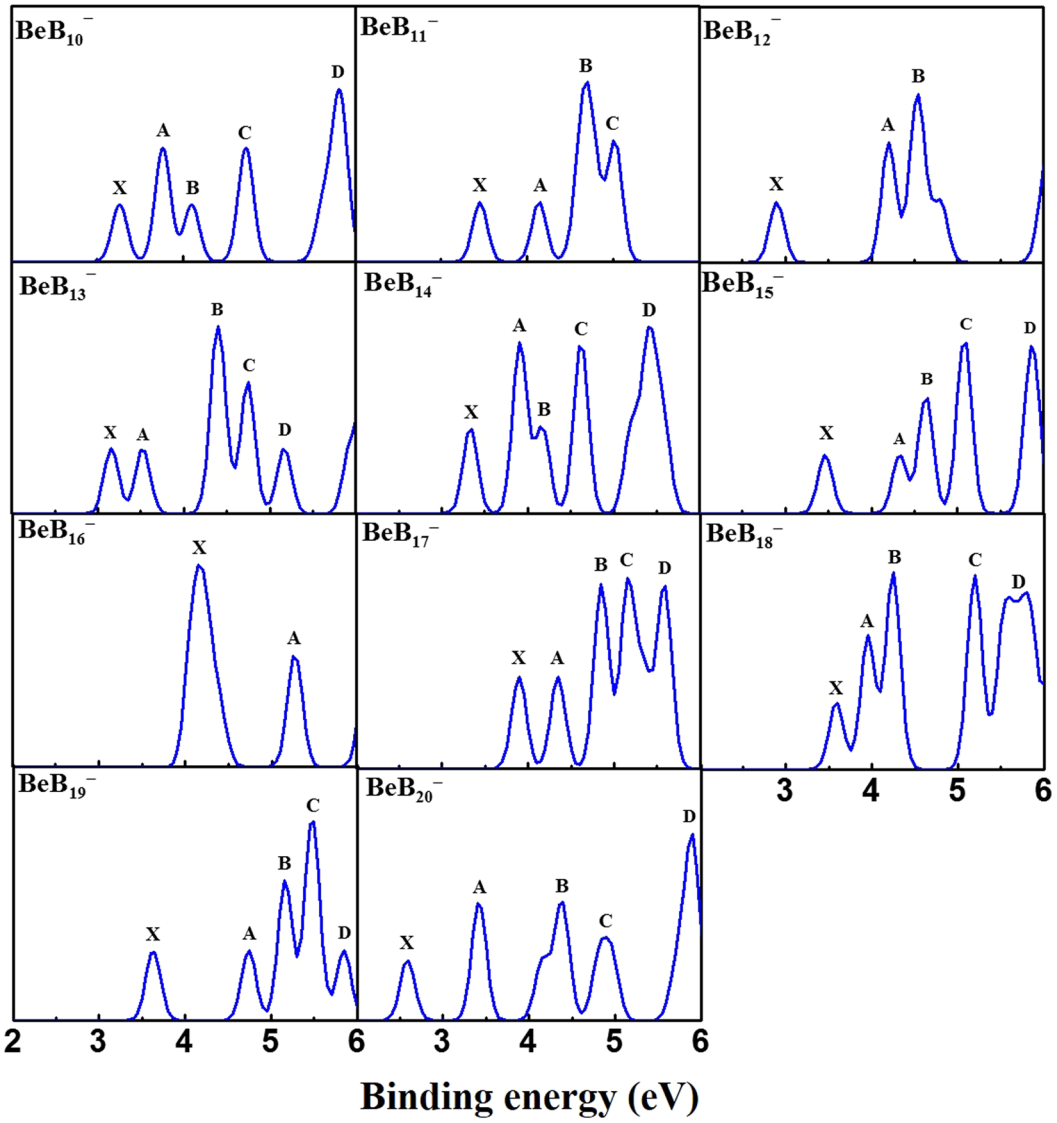
The simulated PES of BeB_*n*_^−^ (*n* = 10–20) clusters.

Photoelectron spectra (PES) analysis, obtained via a TD–DFT approach, is of absolute importance for the assessment of the nature of the determined lowest-energy structures. We simulated the PES of BeB_*n*_^−^ clusters and the results are displayed in Fig. 3. Our group also simulated the PES of some other cluster system using the method^62,63^. The PES pattern of the BeB_10_^−^ possesses five peaks located at 3.26, 3.75, 4.18, 4.75 and 5.77 eV. The PES of BeB_11_^−^ possesses four clear peaks at 3.45, 4.21, 4.59, and 5.01 eV, with B and C peaks forming a broad bond. For BeB_12_^−^, we observe three major peaks at 2.90, 4.21 and 4.50 eV, wherein the double-peak feature (A and B) is prominent and broad. The BeB_13_^−^ PES contains five major peaks at 3.16, 3.49, 4.32, 4.75 and 5.22 eV. The relevant broad bond is found at triple-peak feature consisted of peaks B, C and D. Five peaks are observed for BeB_14_^−^ at 3.33, 3.86, 4.16, 4.63 and 5.45 eV. The peaks A, B and C constitute a relatively wide bond. For BeB_15_^−^ there are five major peaks at 3.46, 4.28, 4.64, 5.06 and 5.82 eV, whereas the BeB_16_^−^ spectrum has only two sparse peaks at 4.08 and 5.25 eV. The well-structure spectrum of BeB_17_^−^ shows five peaks at 3.90, 4.32, 4.79, 5.13 and 5.49 eV, suggesting a greater span triple-peak feature (B, C and D). A crowded spectrum pattern BeB_18_^−^ has five peaks observed at 3.59, 3.98, 4.21, 5.13 and 5.57 eV, with two broad bonds. There are five peaks in the spectrum of BeB_19_^−^ at 3.63, 4.73, 5.13, 5.51 and 5.82 eV, therein an unfitted bond is located at the range between 4.5 to 6.0 eV. The spectrum of BeB_20_^−^ possesses five peaks at 2.59, 3.36, 4.43, 4.85 and 5.79 eV.

### Relative stabilities

We characterize the inherent stability of the BeB_*n*_^0/−^ (*n* = 10–20) clusters by computing the *E*_*b*_ (eV), according to the following formula:

1 $${E}_{b}(Be{B}_{n})=[nE(B)+E(Be)-E(Be{B}_{n})]/(n+1)$$

2 $${E}_{b}(Be{B}_{n}^{-})=[(n-1)E(B)+E({B}^{-})+E(Be)-E(Be{B}_{n}^{-})]/(n+1)$$

The average binding energy (*E*_*b*_) of a cluster is clearly a measure of its thermodynamic stability. An increase in *E*_*b*_ means a higher stability. The value of neutral BeB_*n*_ clusters less than the value of their anionic counterparts in Fig. S1(a), indicating that the anionic clusters feature higher thermodynamically. The trend of the curves for both neutral and anionic are gradually upward indicated that the high thermodynamic stability with the cluster size increases. The second vital physical quantity we take into account here is the Δ^2^*E*. The relevant formulae are

3 $${\Delta }^{{\rm{2}}}E(Be{B}_{n})=E(Be{B}_{n-1})+E(Be{B}_{n+1})-2E(Be{B}_{n})$$

4 $${\varDelta }^{2}E(Be{{B}_{n}}^{-})=E(Be{B}_{n-1}^{-})+E(Be{B}_{n+1}^{-})-2E(Be{B}_{n}^{-})$$

As inferred from Fig. S1(b), both of the neutral and anionic curves show odd-even alteration. The evident peak values generated at *n* = even number, suggest that clusters with the even boron atoms feature higher stability than which with odd boron atoms. Finally, we discuss the HOMO-LUMO energy gap (*E*_*gap*_) which provides a valuable index of the stability of clusters. Large values indicate strong chemical stability. We summarize the *E*_*gap*_ values of the lowest-energy BeB_*n*_^0/−^ clusters in Table 1, and the line chart is displayed in Fig. S1(c). From the latter we can clearly see some apparent local maxima: BeB_11_ and BeB_16_^−^, which means that they feature higher stability than the others. Consequently, based on the above analyses, we can reach a definitive conclusion that the BeB_16_^−^ can seen as a “magic” cluster.

**Table 1:** The calculated electronic states, symmetries, average binding energies (*E*_*b*_, in eV) and energy gaps (*E*_*gap*_, in eV) of BeB_*n*_^0/−^ clusters in the size range of *n* = 10–20.

BeB_*n*_					BeB_*n*_^−^			
*n*	Sta.	Sym.	E_*b*_	E_*gap*_	Sta.	Sym.	E_*b*_	E_*gap*_
10	^1^A′	C_*s*_	4.69	2.93	^2^A″	C_*s*_	5.06	2.81
11	^2^A′	C_*s*_	4.73	3.30	^1^A_1_	C_2*v*_	5.08	2.36
12	^1^A′	C_*s*_	4.87	2.94	^2^A_2_	C_2*v*_	5.17	1.66
13	^2^A″	C_*s*_	4.84	1.74	^3^B_2_	C_2*v*_	5.14	2.17
14	^3^A″	C_*s*_	4.90	2.47	^2^A″	C_*s*_	5.23	2.14
15	^2^A″	C_*s*_	4.93	1.85	^1^A′	C_*s*_	5.20	1.89
16	^1^A′	C_*s*_	4.99	1.77	^2^B_2_	C_2*v*_	5.29	2.93
17	^2^A′	C_*s*_	5.03	2.17	^1^A′	C_*s*_	5.30	2.03
18	^1^A	C_1_	5.08	2.60	^2^A	C_1_	5.32	1.89
19	^2^A	C_1_	5.18	1.97	^1^A	C_1_	5.25	2.39
20	^1^A	C_1_	5.10	2.67	^2^A′	C_*s*_	5.31	2.09

### Chemical banding

**Figure 4:**
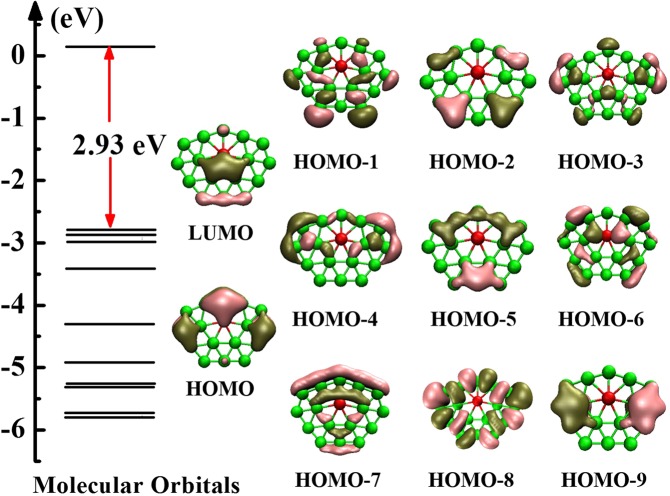
Molecular orbitals for BeB_16_^−^ cluster corresponding to different energy level.

**Figure 5:**
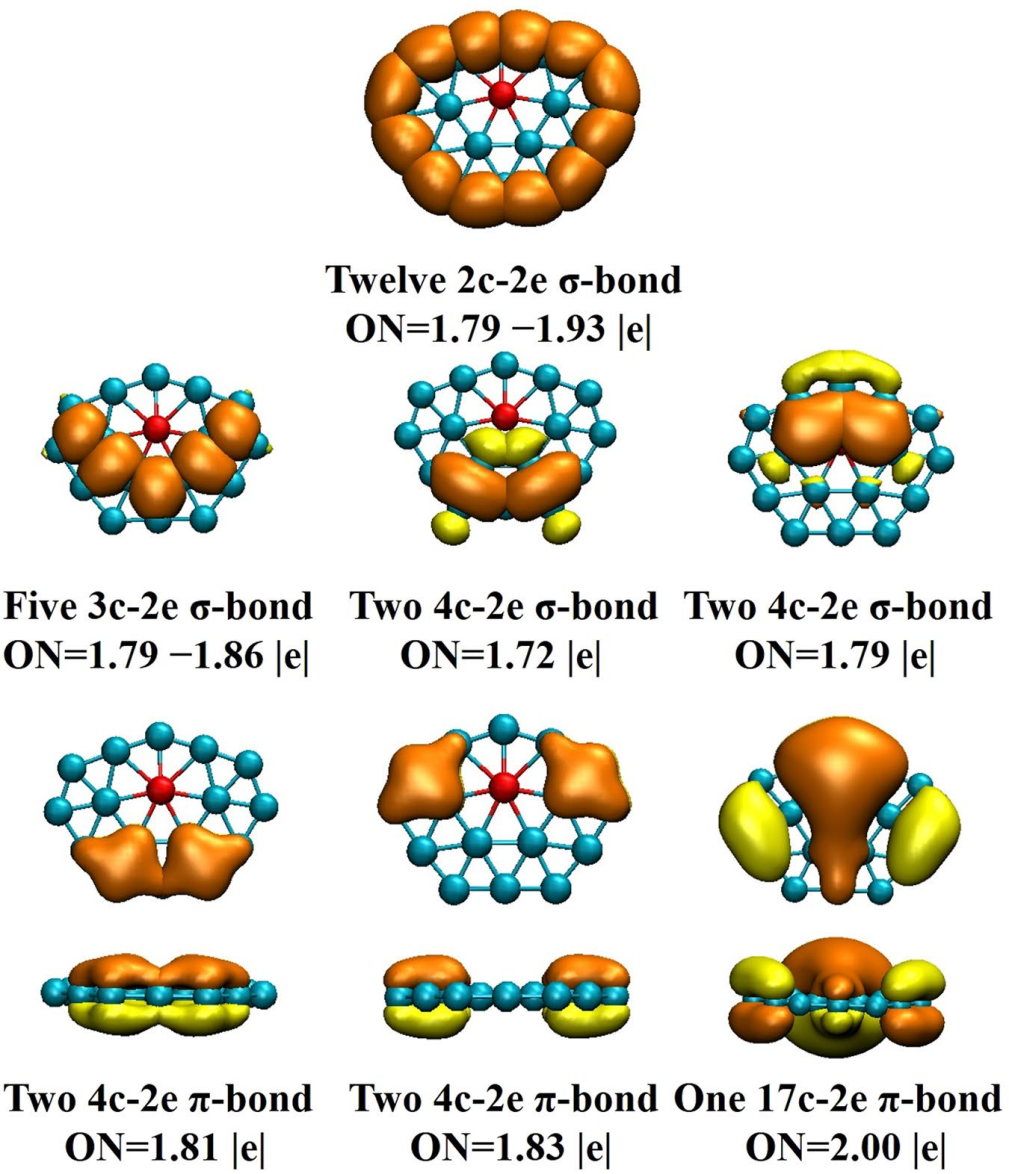
AdNDP analysis of BeB_16_^−^ cluster.

To deeply perceive the bonding nature of BeB_16_^−^ (C_2*v*_ symmetry), we display eleven MO figures for BeB_16_^−^, including one LUMO, one HOMO and nine HOMO-*n* (*n* = 1–9) in Fig. 4 by analyzing the chemical bonding. The LUMO, HOMO, HOMO-2, HOMO-5 and HOMO-9 dominated primarily by π_*p*_ and π_*p*_^∗^ orbitals are a direct interaction 2*p* orbitals of B atoms. The HOMO-*n* (*n* = 1, 4, 8) feature σ_*p*_ and σ_*p*_^∗^ orbitals. The HOMO-*n* (*n* = 3, 6, 7) features σ_*p*_, σ_*p*_^∗^, σ_*sp*_ and σ_*sp*_^∗^ orbitals. AdNDP analysis distributes 51 valence electrons into different regions as reflected by the occupation numbers (ONs) in Fig. 5. We divide it into three sets. The first set consists of twelve 2c-2e (1.79–1.93 |e|) localized σ-bonds. The second set consists of nine delocalized σ-bonds, which are five 3c-2e (1.79–1.86 |e|), two 4c-2e (1.72 |e|), and two 4c-2e (1.79 |e|). The five delocalized *π*-bonds in last set involving two 4c-2e (1.81 |e|), two 4c-2e (1.83 |e|) and one 17c-2e (2.00 |e|). It is worth nothing that the ON of the 17c-2e *π*-bonds maintain ON of 2.00 |e|. All values of the ONs listed above ranging from 1.72–2.00 |e| are approaching the ideal value 2.00 |e|, which means that the results we calculate is fairly credible. Furthermore, the ten *π* electrons conform to the 4*n* + 2 rule (*n* = 2), indicating the BeB_16_^−^ cluster possesses *π*-aromaticity, which result to the robust relative stability for BeB_16_^−^ cluster.

The Wiberg bond index of BeB_16_^−^, showed in Fig. S2(a), indicate that the bond orders values of B-B (0.13–0.35) greater than the Be-B (0.06–0.11). For Fig. S2(b), the B-B bond lengths (1.54–1.80 Å) are shorter than Be-B bond lengths (1.85–2.03 Å). The results of bond orders and bond lengths show that the peripheral B-B bonds are stronger than the inner Be-B bonds. We have also performed the NPA (natural charge of atom) calculations of BeB_*n*_^0/−^ in Fig. S3 indicate that electron transfer from Be atom to boron fragment. The NPA data of BeB_*n*_^0*/*−^ (*n* = 10–20) clusters are summarized in Table S1. From what has been discussed, we come to the conclusion that the B-B *σ*-bonds and the aromaticity decide the high stability of BeB_16_^−^ cluster. It is worth noting that due to planar structure and chemical bonding characteristics of BeB_16_^−^ cluster, also inspired by fascinating prospect of two-dimensional monolayer metallo-borophene^4^, we successfully build a schematic of possibility of metallo-borophene (not optimized) based on BeB_16_^−^ unit cluster presented in Fig. S6 of Supplementary Information, which indicated the BeB_16_^−^ cluster is a potential motif for metallo-borophene.

## Conclusions

In summary, the ground-state BeB_*n*_^0/−^ (*n* = 10–20) structure obey the evolution rule: quasi-planar to 3D cage-like or plate-like structures, which the doped Be atom contributed to the plane or quasi-plane structures. We hope that the simulated PES can provide valuable guidance for future research on BeB_*n*_ clusters and borophene. Based on the relative stability analysis, the BeB_16_^−^ cluster characterized by enhanced stability is clearly a “magic” cluster. Chemical bonding analysis indicated that BeB_16_^−^ cluster adapt *π*-aromaticity and the strong interaction of B-B σ-bonds which is deemed as the dominant reasons for the inherent stability of BeB_16_^−^ cluster. The planar BeB_16_^−^ cluster may serve as a motif for the design of a new boron-based functional material to complement the metallo-borophene effort for synthetic 2D materials development. Our present findings on Be-doped boron clusters should provide valuable information for further explorations of novel cluster architectures.

## Computational Methods

We used the CALYPSO code to search the BeB_*n*_^0/−^ (*n* = 10–20) clusters. The global explorations of Be-doped boron cluster system was implemented by utilizing particle swarm optimization (PSO) algorithm^64–66^. The effectiveness of this structural prediction method, has been successfully tested on the identification of ground-state structures of various systems^67–69^. To ensure high efficiency in structure predicting, we proceeded to 50 generations for each size, where each generation contains 30 structures. PSO algorithm produces sixty percent of the structures and the rest is generated randomly. The top fifty low-lying isomers were reoptimized with PBE0^70^ functional and 6–311 + G(d)^71^, as performed via Gaussian 09 package^72^. The PES of Be-doped boron clusters was simulated utilizing TD-DFT method^73^. We then analyzed chemical bonding of BeB_16_^−^ cluster relying on the NBO and AdNDP methods^74^ at the PBE0/6-311 + G(d) level to display valuable insights into the nature of the bonding by using Multiwfn^75^. The bond orders, bond lengths and NPA are also computed by using the same basis set and method.

## Supplementary information

Supplementary_Information
