# Supplementary material for: Probing the structure and electronic properties of beryllium doped boron clusters: A planar BeB16− cluster motif for metallo-borophene
Source: Sci Rep. 2019 Oct 7;9:14367. doi: 10.1038/s41598-019-50905-7 (PMC6779740; doi:10.1038/s41598-019-50905-7)
Supplement: Supplementary file 1 — Supplementary_Information [file 41598_2019_50905_MOESM1_ESM.doc]

**Probing the structure and electronic properties of beryllium doped boron clusters: A planar BeB_16_^−^ cluster motif for metallo-borophene**

Dongliang Kang^1^, Weiguo Sun^1^, Hongxiao Shi^1^, Cheng Lu^2^, Xiaoyu Kuang^1,∗^, Bole Chen^1^, Xinxin Xia^1^, and George Maroulis^3,∗^

^1^Institute of Atomic and Molecular Physics, Sichuan University, Chengdu 610065, China

^2^School of Mathematics and Physics, China University of Geosciences (Wuhan), Wuhan 430074, China

^3^Department of Chemistry, University of Patras, GR-26500 Patras, Greece

^*^Correspondence author. E-mail: scu_kuang@163.com (Xiaoyu Kuang), and maroulis@upatras.gr (George Maroulis)

***Table*** ***S1****.* *Calculated NPA (e) for BeB_n_^0/−^ (n = 10−20) clusters.*

| *n* | NPA (e) |  |
| --- | --- | --- |
|  | BeB*_n_* | BeB*_n_^－^* |
| 10 | 0.51 | 0.40 |
| 11 | 0.70 | 0.74 |
| 12 | 0.38 | 0.40 |
| 13 | 0.76 | 0.85 |
| 14 | 0.64 | 0.57 |
| 15 | 0.86 | 0.89 |
| 16 | 0.57 | 0.57 |
| 17 | 0.74 | 0.61 |
| 18 | 0.82 | 0.66 |
| 19 | 0.80 | 0.65 |
| 20 | 0.90 | 0.80 |
|  |  |  |


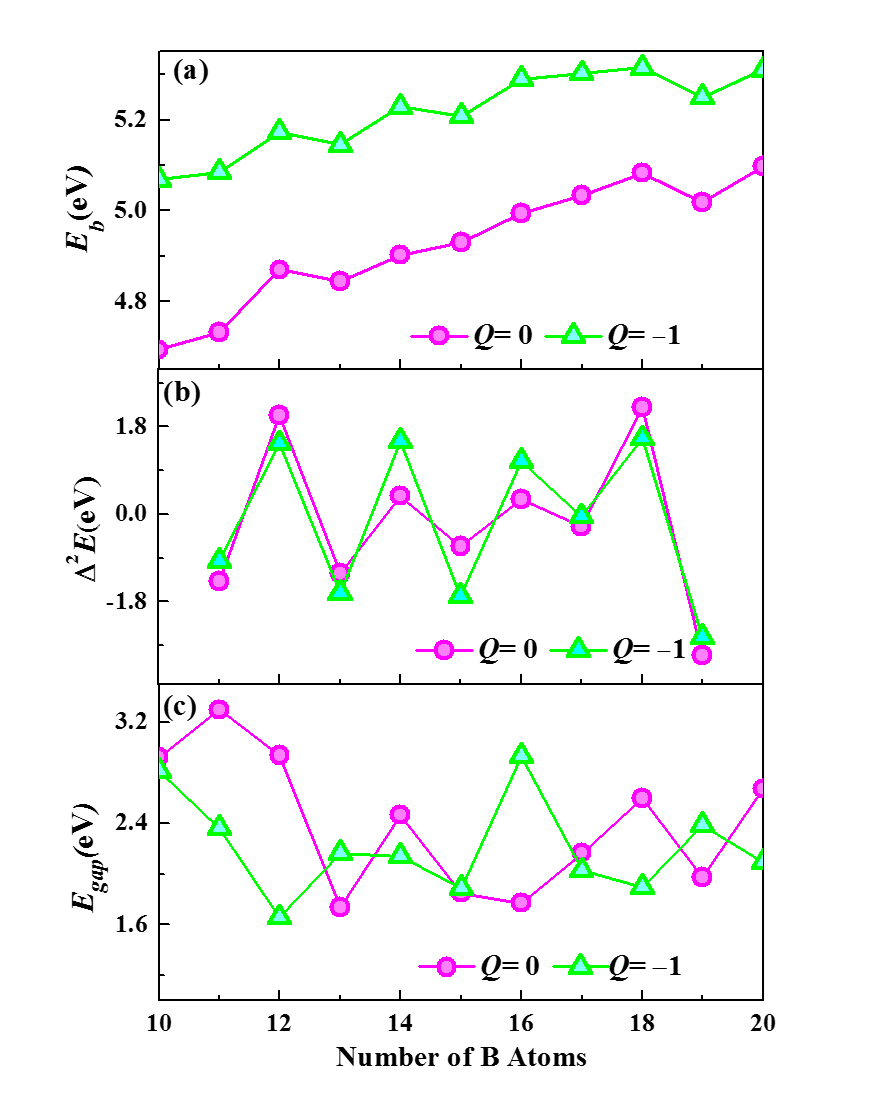


**Figure S1**. (a) The average binding energies *E_b_*, (b) second-order energy differences *Δ^2^E*, and (c) HOMO-LUMO energy gaps *E_gap_* for lowest-energy BeB*_n_*^0/−^ clusters in the size range of *n* = 10−20.


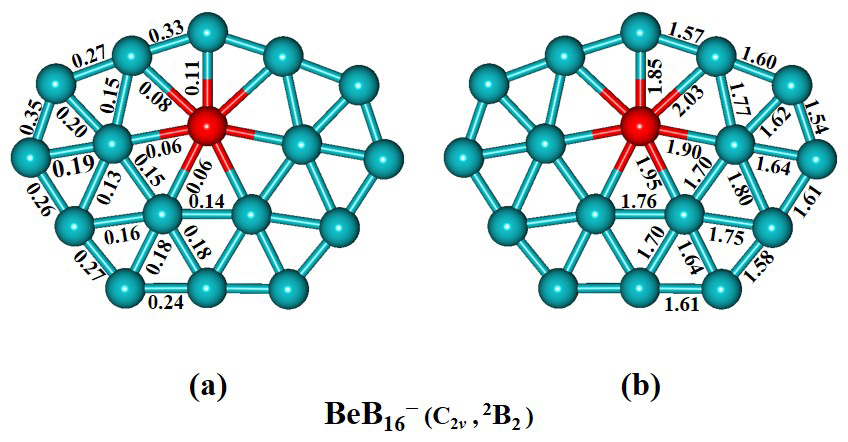


**Figure S2**. Structure details of lowest-energy of BeB_16_^−^ at PBE0/6-311+G(d) with their point group symmetries and electronic states. (a) The numbers are the Wiberg bond index (WBI) of representative chemical bonds. (b) The numbers are representing the corresponding bond lengths.


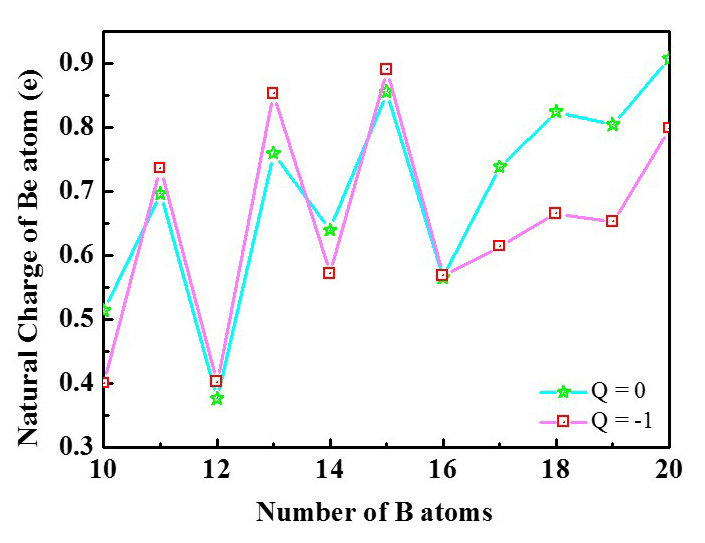


**Figure S3**. The natural charge of Be atom in the lowest total energy states of the BeB*_n_*^0^*/*^−^ (*n* = 10−20) clusters as a function of the number of boron atoms.


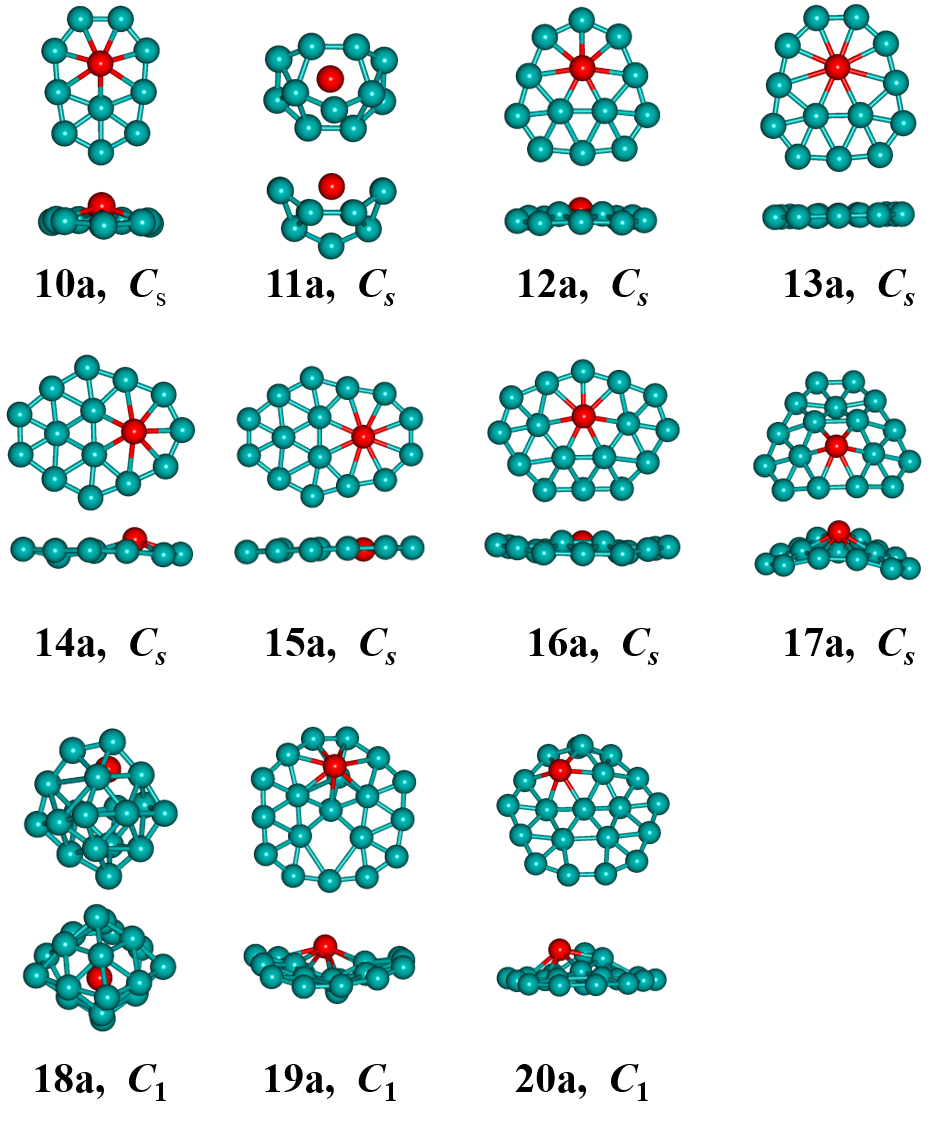


**Figure S4**. The front view and side-on view of lowest-energy structures BeB*_n_* (*n* = 10−20) clusters.


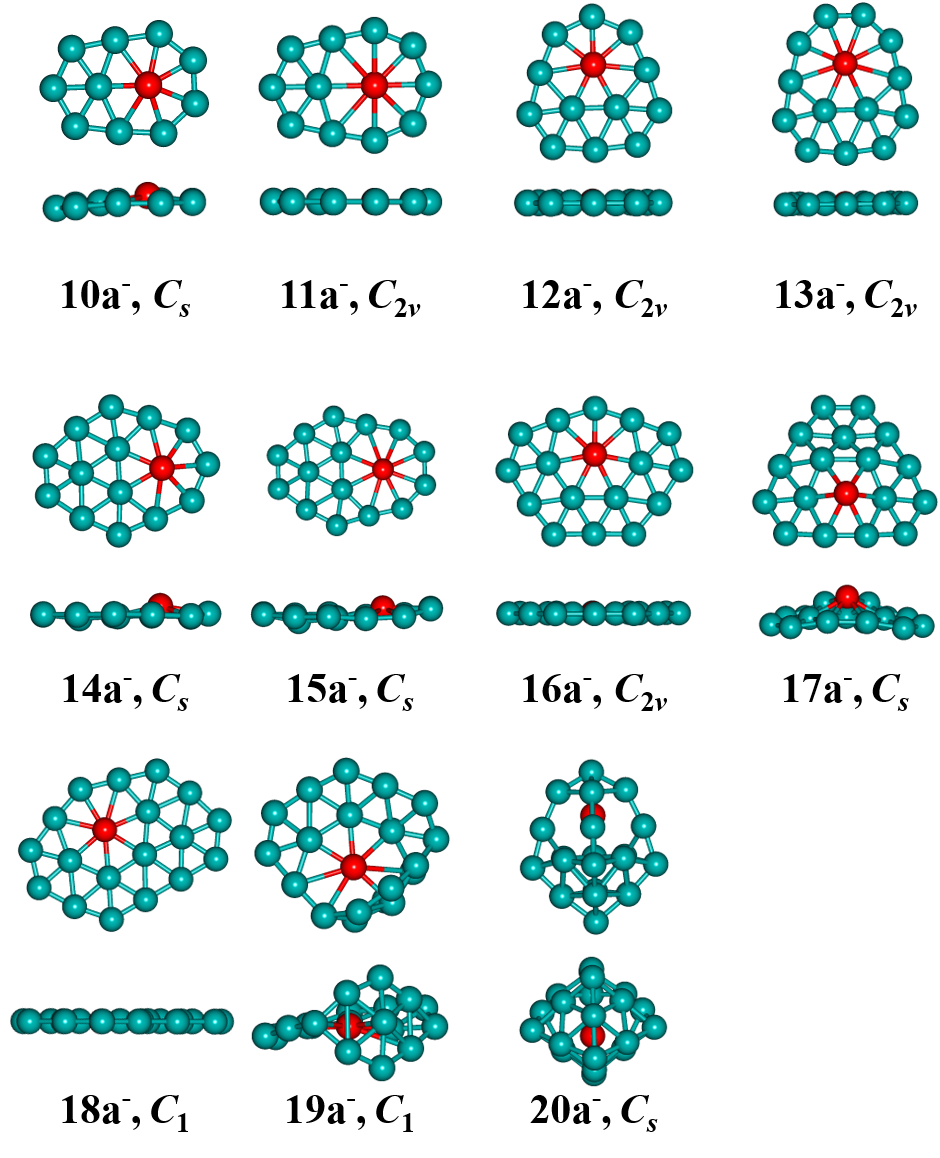


**Figure S5**. The front view and side-on view of lowest-energy structures BeB*_n_*^−^ (*n* = 10−20) clusters.


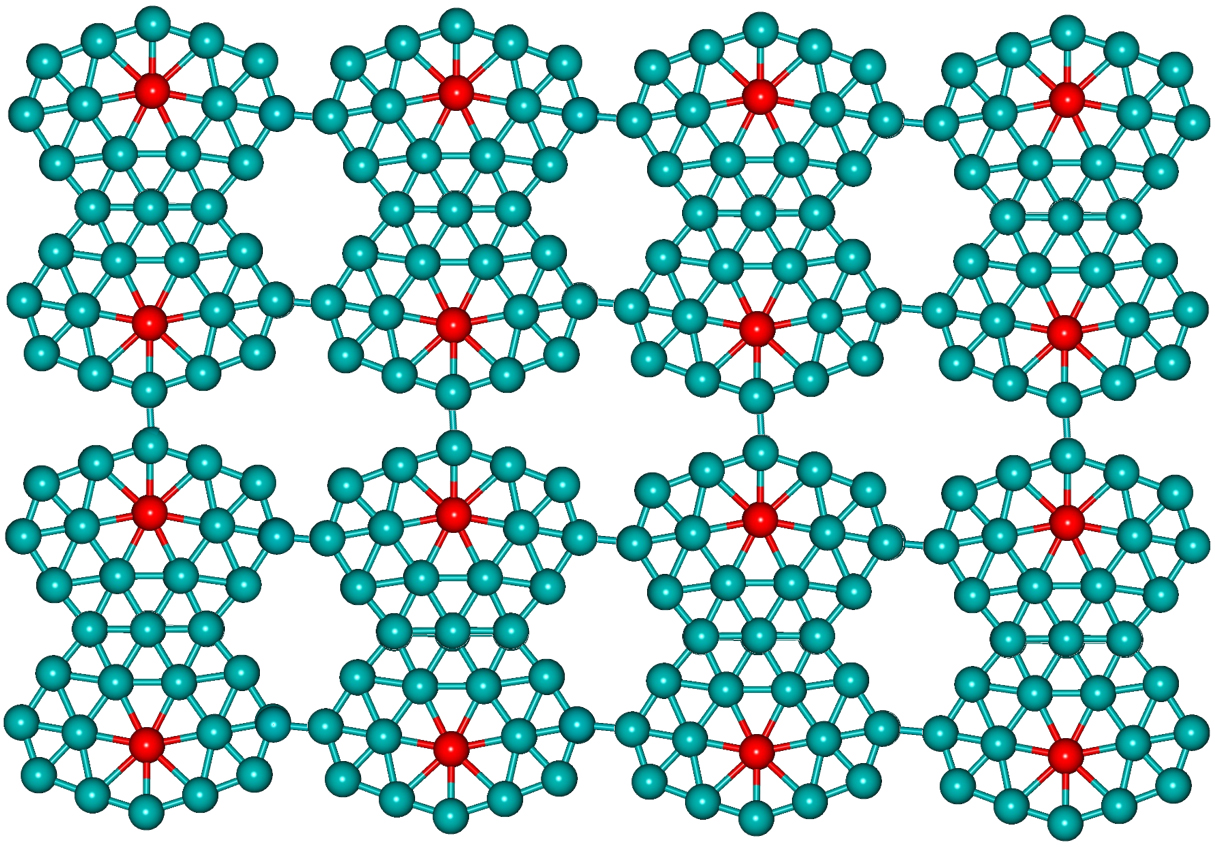


**Figure S6.** A possible metallo-borophene layer structure (not optimized) schematic based on BeB_16_^−^ unit.
